# A Between Ethnicities Comparison of Chronic Obstructive Pulmonary Disease Genetic Risk

**DOI:** 10.3389/fgene.2020.00329

**Published:** 2020-04-21

**Authors:** Jungsoo Gim, Jaehoon An, Joohon Sung, Edwin K. Silverman, Michael H. Cho, Sungho Won

**Affiliations:** ^1^Department of Biomedical Science, Chosun University, Gwangju, South Korea; ^2^Graduate School of Public Health, Seoul National University, Seoul, South Korea; ^3^Department of Public Health Sciences, Graduate School of Public Health, Seoul National University, Seoul, South Korea; ^4^Interdisciplinary Program of Bioinformatics, Seoul National University, Seoul, South Korea; ^5^Institute of Health and Environment, Seoul National University, Seoul, South Korea; ^6^Channing Division of Network Medicine and Division of Pulmonary and Critical Care Medicine Division, Brigham and Women’s Hospital, Harvard Medical School, Boston, MA, United States

**Keywords:** COPD, ethnicity-specific, SNP heritability, susceptible loci, BLUP-filtered SNP, genetic prediction, ethnicity difference

## Abstract

Heterogeneity of lung function levels and risk for developing chronic obstructive pulmonary disease (COPD) among people exposed to the same environmental risk factors, such as cigarette smoking, suggest an important role of genetic factors in COPD susceptibility. To investigate the possible role of different genetic factors in COPD susceptibility across ethnicities. We used a population-stratified analysis for: (i) identifying ethnic-specific genetic susceptibility loci, (ii) developing ethnic-specific polygenic risk prediction models using those SNPs, and (iii) validating the models with an independent dataset. We elucidated substantial differences in SNP heritability and susceptibility loci for the disease across ethnicities. Furthermore, the application of three ethnic-specific prediction models to an independent dataset showed that the best performance is achieved when the prediction model is applied to a dataset with the matched ethnic sample. Our study validates the necessity of considering ethnic differences in COPD risk; understanding these differences might help in preventing COPD and developing therapeutic strategies.

## Introduction

Patients with chronic obstructive pulmonary disease (COPD) suffer from decreased expiratory airflow, increased airway resistance, and hyperinflation. Although its association with other environmental risk factors has been previously reported, cigarette smoking has been identified as the major environmental risk factor for COPD development (Lin et al., 2008). However, not all smokers develop COPD, and longitudinal lung function decline among those with similar smoking and exposure histories can vary remarkably. In addition, a recent multi-ethnic study indicated substantial geographic differences in COPD characteristics, which could be genetic or environmental (Kim et al., 2017). Together with previous reports (Kirkpatrick and Dransfield, 2009; Hansel et al., 2013; Kamil et al., 2013), these observations suggested an important role of genetic and ethnic differences in COPD development.

A number of studies have been performed to elucidate genetic roles in COPD susceptibility, ranging from twin and pedigree-based studies of familial aggregation to case-control genetic association analyses (Redline et al., 1989; Sandford et al., 1997; Silverman et al., 1998; Cho et al., 2010, 2012, 2014; Ingebrigtsen et al., 2010; Hardin and Silverman, 2014; Benyamin et al., 2017). Although these have successfully identified several significant COPD-susceptibility loci, no attempt has yet been made to investigate the likelihood of different genetic background-associated ethnic differences in the risk of COPD. One of the major challenges of multi-ethnic genomic studies is the lack of proper multi-ethnic data (Bustamante et al., 2011). Although the COPDGene project includes a large number of non-Hispanic White and non-Hispanic African American cases and controls, it has no Asian samples (Regan et al., 2010). On the other hand, a large number of Asian samples are publicly available from the KARE cohort study (Cho et al., 2009), that includes a limited number of patients with COPD. Another important challenge in multi-ethnic studies is spurious associations. A number of factors, such as cryptic population and confounding bias, which can produce spurious associations (Price et al., 2006; Yang et al., 2011b; Bulik-Sullivan et al., 2015), or polygenicity, which can cause substantial genomic inflation (Yang et al., 2011b), should be accounted for when conducting larger studies. Moreover, there is limited, but important, evidence of ethnic heterogeneity as a genetic risk in COPD (Kirkpatrick and Dransfield, 2009; Silverman and Sandhaus, 2009; Kamil et al., 2013), suggesting potential between ethnicities variations related to specific genetic risk loci, also referred to as, “between ethnicities polygenicity” of COPD.

In this study, we aim to address the issue of between ethnicities differences of genetic risk and polygenicity in the development of COPD by evaluating ethnicity-specific polygenic risk modeling in COPD risk prediction using available datasets. We performed a stratified analysis under the following assumptions: COPD is a complex polygenic disease and the polygenicity can vary depending on ethnicity. We investigated the genotype datasets of African Americans (AA) and non-Hispanic Whites (NHW) from COPDGene (Regan et al., 2010), and of East Asians (EA) from KARE (Cho et al., 2009) project. We first observed different SNP-related heritability of COPD among ethnicities, then identified ethnicity-specific genetic susceptibility loci (SNPs), filtered by the best linear unbiased prediction (BLUP) from linear mixed models. Subsequently, we developed three different ethnicity-specific polygenic risk prediction models incorporating many SNPs using penalized regression techniques. We showed that models with a known environmental risk factor, i.e., cigarette smoking, combined with ethnicity-specific SNPs, can improve prediction performance. Finally, the validity of ethnicity-specific modeling was examined using an independent dataset from the MESA project (Bild et al., 2002).

Throughout this study, polygenic risk prediction was used to show the importance of the work. The importance is twofold: first, it presents the possibility of genomic prediction in clinical practice; and second, it shows the necessity of considering ethnic-wise polygenic nature of COPD development. Since COPD is a progressive debilitating lung condition with impact on both morbidity and early mortality, predicting those at increased risk of developing COPD can allow for implementation of interventions which may not only prevent COPD developing, but may also help preserve lung function and quality of life in those who do go on to develop COPD. All prediction models might differ in predictors used, outcome definitions, and ethnicities from which they were developed. The models predicting current status of COPD development generally perform well with clinical symptoms included. However, predicting future COPD risk, which has the most clinical usefulness, is particularly difficult because of lack of proper predictors. Here, we show that the inclusion of more SNPs with larger effect but no statistical (genome-wide) significance could improve the prediction ability of the models.

## Materials and Methods

### Preparing Multi-Ethnic Dataset

To compare multi-ethnic parameters as a risk factor in COPD, three different ethnic datasets were used to build polygenic prediction models. Genotype and phenotype datasets of AA and NHW were obtained from the COPDGene project (Regan et al., 2010) and those of EA were provided by the KARE project (Cho et al., 2009). To validate the polygenic risk prediction models, we used a dataset from the MESA project. Because of our limiting accessibility, a part of MESA datasets (NHW) was only available. To analyze the complete dataset, individuals with missing values in their covariates (age, sex, current smoking, pack-years of smoking, and family history of COPD) or genetic information (SNPs of interest) were discarded from further analyses.

### Genotype Imputation

Quality control (QC) and genotype imputation were performed for KARE (352,228 SNPs in 8,842 individuals) and MESA (909,622 SNPs in 2,255 NHW subjects). SNPs for which the missing call rate was larger than 5%, minor allele frequency (MAF) was less than 5%, and *p*-value of Hardy–Weinberg equilibrium (HWE) test was less than 1e-05, were removed. Participants with missing call rate above 5% or sex inconsistency were also excluded. After QC, 310,515 SNPs in 8,773 individuals in KARE and 679,760 SNPs in 2,255 subjects in MESA were retained. The imputation method applied in this work is the combination of SHAPEIT (Delaneau et al., 2011) and IMPUTE2 (Howie et al., 2009), which shows generally higher performance in a recent benchmark paper (Roshyara et al., 2016). SHAPEIT2 v2.r837 and IMPUTE2 version 2.3.2 were used for data pre-phasing and genotype imputation. Each chromosome was split into small chunks with length of 3 Mb for imputation, and each output was concatenated into single genotype data with whole chromosome. Internal buffer regions of 1 Mb on either side of chunks also used in every imputation analysis. The haplotypes data in phase 3 of the 1000 Genomes Project were used as the reference panel. Imputed SNPs with information metric in IMPUTE2 below 0.5, in which a very small number of genotypes are called with a poor concordance rate, were excluded from this study.

### SNP Screening

To select an effective short list (e.g., with large effect size) of SNPs for a prediction model, we evaluated the BLUP of each SNP. From using $Y∼M\,V\,N\,\left(Z\,\beta +\sigma_{\text{g}}^{2}\,G\,G^{\prime }+\sigma^{2}\,I\right)$, where *Z* and *G* denote demographic variables with fixed effects and a genotype matrix with random effects in the training set, respectively. The genotype variance $\sigma_{\text{g}}^{2}$ and residual variance σ^2^ can be solved using restricted maximum likelihood (REML). The BLUP of each SNP is defined as $G^{\prime }\,K^{-1}\,\left(Y-Z\,\hat{\beta }\right)/{\hat{\sigma }}_{\text{g}}^{2}$, where *K* is the genetic relationship matrix (GRM) estimated from SNPs. SNP-wise BLUP can be calculated using GCTA with –blup-snp option (Yang et al., 2011a). To build the prediction models, SNPs with top *p* (*p* = *100, 500, 1000, 5000*, and *10000)* were selected based on the largest absolute BLUP value or the smallest *p*-value. For the evaluation of BLUP, we modeled FEV1 with each SNP as a random effect and with age, sex, height, and pack-years as fixed effects using GCTA (Yang et al., 2011a). Similarly, *p*-values were evaluated from the linear regression model using PLINK (Purcell et al., 2007).

### Building Polygenic Prediction Model Using Penalized Regression Methods

Let *X*_i_ = (*Z*_i_,*G*_i_) and *Y*_i_ be a covariate vector and a dichotomous COPD status for subject *i*. We further denote *G*_il_ and *Z*_im_ as coded genotypes of the *l*^th^ SNP selected from BLUP screening and the *m*th clinical covariate, respectively. The *r*-dimensional coefficient vector β consists of *p* genetic variants and *q* clinical variables. Under this model, β can be estimated by minimizing the penalized negative log-likelihood:

(1) $$\frac{1}{n}\,\sum_{i=1}^{n}\left\{-Y_{i}\,X_{i}^{\prime }\,\beta +l\,o\,g\,\left(1+\exp\left(X_{i}^{\prime }\,\beta \right)\right)\right\}+\sum_{l=1}^{p}J_{\lambda }\,\left(\left|\beta_{l}\right|\right)$$

where *J*_λ_ is a penalty function and λ is a vector of a tuning parameter that can be determined by a search on an appropriate grid. Note that only genetic variants are penalized with Lasso (Tibshirani, 1996), Ridge (Hoerl, 1970), and Elastic Net (EN) (Zou and Hastie, 2005) penalty functions. All analyses were performed on R software with *glmnet* (Friedman et al., 2010) R package.

### Evaluating Variability Based on Each Variable in Penalized Logistic Regression

To estimate variability of each variable in the penalized regression model, we used the deviance, calculated by comparing the predicted and true phenotypes in a test dataset, as seen in Gim et al. (2017). Specifically, we built the prediction model with a training set and applied it to predict the phenotypes of test samples. The deviance was obtained by comparing the predicted phenotypes and true phenotypes for those samples. If we denote the predicted and true phenotypes by $\hat{\mu_{i}}$ and *Y*_*i*_, respectively, deviance would be defined as

(2) $$\Delta =\sum_{i}\left\{Y_{i}\,\log\frac{Y_{i}}{\hat{\mu_{i}}}+\left(1-Y_{i}\right)\,\log\frac{1-Y_{i}}{1-\hat{\mu_{i}}}\right\}$$

We used 5-fold cross validation and the deviances for all subjects were evaluated by summing all deviances in the test set. Based on Eq. 2, we defined the variability explained by the current model (*Δ*_*F*_) using McFadden’s R^2^ (McFadden, 1974):

$$1-\frac{\Delta_{F}}{\Delta_{0}}\times 100$$

where *Δ*_*0*_ is the deviance of the null model. The variability that remained unexplained by the full model may be obtained by 1- McFadden’s. If we denote the reduced model, whose *i*th element is excluded, by Δ_*i*_, and further define the relative deviance explained by the *i*th variable as

(3) $$1-\frac{\Delta_{F}}{\Delta_{0}}\times 100-\left(1-\frac{\Delta_{i}}{\Delta_{0}}\times 100\right)=\frac{\Delta_{i}-\Delta_{F}}{\Delta_{0}}\times 100$$

Eq. 3 would represent the relative deviance explained by the *i*th variables out of total variability.

## Results

### Overview of the Work

**FIGURE 1:**
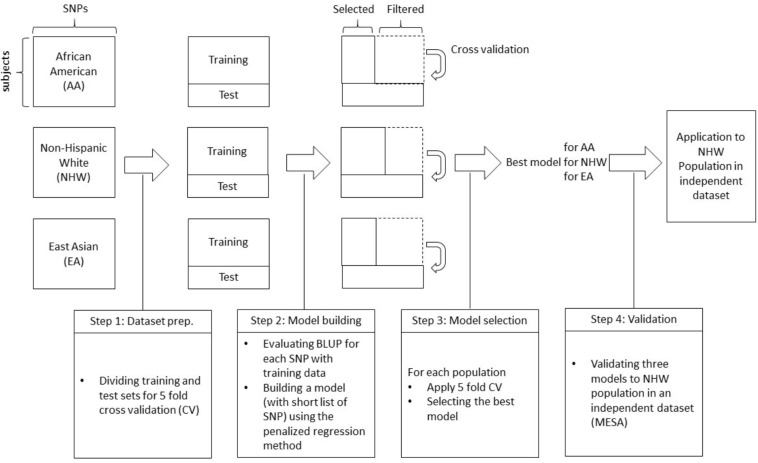
A schematic diagram of the proposed work.

We briefly outline the analyses performed in this work (Figure 1).

• Step 0: Genotype and phenotype information from three different ethnic groups were collected from two large study projects.
• Step 1: To perform 5-fold cross-validation for each ethnic group, each dataset is divided into five different subsets, one of which is used as a test set and the other four are used as training sets. Based on training dataset, the BLUP was calculated and sorted by largest absolute BLUP values.
• Step 2: Using the training set, SNPs are pre-screened with BLUP criteria, i.e., SNPs with the top-*p* largest absolute BLUP value are selected. Here, we considered *p* = 100, 500, 1000, 5000, 10000, 15000, and 20000. Based on this list, a prediction model is built with training dataset using penalized regression methods (Lasso, Ridge, and Elastic-Net) and validated on test data.
• Step 3: Tuning parameters for each penalized regression are selected with a nested cross-validation scheme. For each training set (four out of five), data is divided into 10 sub-datasets again, and for different choices of tuning parameters, the prediction model is obtained with the other nine sub-datasets. The area under the curve (AUC) is then calculated with the remaining sub-dataset, and tuning parameters that result in the largest AUC are finally chosen to generate the final prediction model for the first CV set (out of five). Then the final prediction model is applied to the test set for first CV set. These steps are repeated for the remaining four CV sets to identify the best performing model for each population.
• Step 4: The best model for each population is applied to an independent dataset to validate the best models.

### Characteristics of Study Samples

Datasets were obtained in previous studies with different designs: case/control study and cohort study. Note that the ratio of COPD cases in EA population is much smaller than those in AA and NHW. All of the EA sample used for The KARE project are from the prospective epidemiological community-based cohorts in Korea and thus the number of patients with COPD is limited. Because of the distinct differences of genotype platforms used, the number of genotyped SNPs available differed in each ethnic group. As the NHW and AA were genotyped using the same platform, we performed imputation in the EA and selected 582,758 SNPs that overlapped among the three groups (AA, EA, and NHW). A brief summary of the datasets used in this study is shown in Table 1.

**TABLE 1:** Baseline characteristics of study samples.

**Project**	**COPDGene**				**KARE**	
**Ethnicity**	**African American**		**Non-hispanic Whites**		**East Asian**	
**Disease**	**COPD**	**Controls**	**COPD**	**Controls**	**COPD**	**Controls**
Sample size	827	1797	2825	2543	725	7253
	2624		5368		7978	
Sex Male/Female	456/371	1033/764	1574/1252	1255/1288	536/189	3175/4078
Age Mean (SD)	59 (8)	53 (6)	65 (8)	59 (9)	58 (8)	51 (9)
Pack-Years Mean (SD)	42 (23)	36 (20)	56 (28)	38 (20)	22 (21)	8 (15)
The number of overlapping SNPs/genotyped SNPs	582,758/713,772		582,758/646,125		582,758^impute/^304,245	
The number of overlapped SNPs without NAs	582,758		582,758		310,703	

### Ethnicity-Specific SNPs and Their Overlaps

Heritability estimates evaluated in previous studies using COPDGene datasets indicated that a substantial proportion of heritability in COPD-related traits, such as FEV1 and FVC, is explained by genome-wide SNPs (Zhou et al., 2013). To observe what fraction of heritability of COPD can be explained by the additive effects of common variants, we evaluated the genetic heritability of FEV1, a variable in continuous scale used to define COPD, with or without smoking status (never-smoked, ex-smoking and smoking) adjusted. Each ethnic group showed marked differences in both total SNP heritability (Table 2) and relative chromosomal SNP heritability (Supplementary Figure S1). Among three different ethnic groups, NHW showed the highest heritability of 41.4%. While AA showed slightly smaller value of 34.9%, EA showed the smallest fraction of FEV1 explained (16.4%). According to previous studies, not only total SNP heritability, but the relative chromosomal SNP heritability, defined as chromosomal proportion of total SNP heritability, also showed a different pattern among ethnicities. For instance, unlike NHW and AA, the heritability in chromosomes 4, 18, and 22 for EA was almost zero (Supplementary Figure S2). The effect of smoking status on heritability estimates was also different among ethnicities, possibly suggesting different genetic roles against smoking in lung function (Table 2).

**TABLE 2:** Prediction with clinical variables (in AUC).

**Models**	**Variables**	**African American**	**East Asian**	**Non-hispanic Whites**
Logistic	Age, Sex	0.732 (0.0374)	0.774 (0.0293)	0.670 (0.0148)
	Age, Sex, Pack-years	0.733 (0.0037)	0.784 (0.0248)	0.746 (0.0120)
	Age, Sex, Family history	0.735 (0.0393)	0.774 (0.0282)	0.679 (0.0177)
	Age, Sex, Pack-years, Family history	0.736 (0.0382)	0.784 (0.0240)	0.750 (0.0126)

*^∗^Parentheses indicates standard deviation of the AUC.*

Since a substantial proportion of heritability was also explained by available SNPs in our study samples (Supplementary Table S1 and Supplementary Figure S1), we analyzed whether there is an ethnic difference in genetic susceptibility loci associated with COPD. We first prioritized SNPs for each ethnic group, based on the BLUP and *P*-value criteria described in Methods, and selected the top 20,000 SNPs for each ethnic group [Supplementary Material containing their detailed statistics and reproducible R script are available either through “figshare” https://figshare.com/s/697ad5a1e4a3d42d413f (DOI: 10.6084/m9.figshare.8246075) or upon request] To observe genetic tendency among the ethnic groups, we counted the number of overlapped SNPs among the ethnic groups.

**FIGURE 2:**
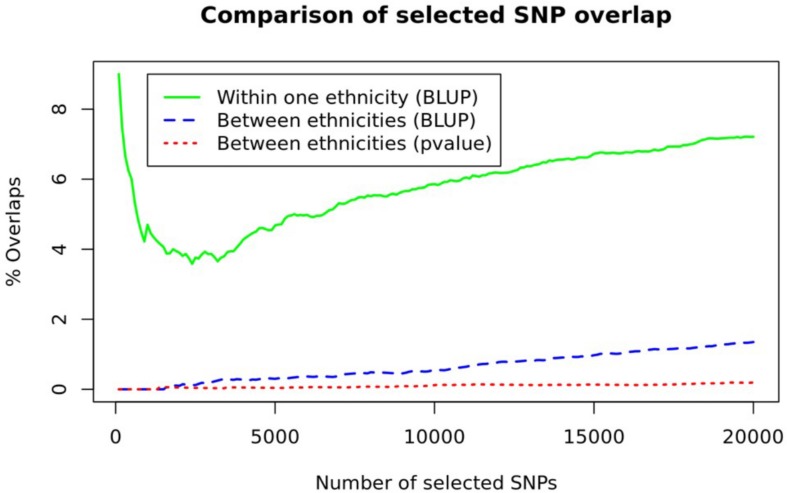
A comparison of SNP overlaps. The number of overlapping SNPs between ethnicities (blue dashed line and red dotted line) and within one ethnicity, obtained from three randomly separated NHW samples (green line), are depicted.

As can be seen in Figure 2, the proportion of overlapping SNPs among the ethnic groups was about 1% or less (Figure 2 and Supplementary Figure S2). Between BLUP and *p*-value criteria, more SNPs prioritized with the BLUP (blue dashed line in Figure 2) were overlapped compared to those prioritized with the *p*-value (red dotted line in Figure 2). To appreciate the amount of between ethnicities difference, we estimated within one ethnicity difference by observing the overlapping SNPs from three randomly separated NHW ethnic groups (green solid line in Figure 2). The proportion of overlapping SNPs in within one ethnicity was seven times higher than that in between ethnicities groups. These results suggest ethnicity-specific prediction models, incorporating ethnicity-specific SNPs, were appropriate for consideration. Note that the overlapping proportions shown in Figure 2 are far smaller than those obtained using CV datasets (45–56%) for each ethnic group (Supplementary Figure S3).

### Prediction Performance (Internal Validation of Each Model)

Prior to building an ethnicity-specific model, we first modeled COPD based on a set of non-genetic markers associated with the disease. We incorporated age, sex, pack-years of smoking, and family history of parents based on questionnaire into the prediction model for COPD risk (result shown in Table 2). From three different ethnic groups, four different models were built with different combinations of predictors. With age and sex as a baseline, predictive power (measured in AUC) increased in all ethnic groups when pack-years of smoking was additionally considered. Note that for the AA model, AUC increase was not distinct but the 10-fold decrease in standard deviation of the AUC was observed when pack-year was incorporated. Unlike pack-years, the inclusion of family history had variable effect. While AUC was slightly increased in AA, it remained unchanged in EA, and decreased in NHW. It was worth noting that AA shows rather robust performance, regardless of inclusion of variables.

The highest absolute AUC for each ethnic group was observed with the inclusion of all the variables. However, family history was not considered further owing to its inconsistent pattern among populations. Such an inconsistency may be due to poor accuracy of self-reports of family history in the dataset, leading to inaccurate estimates of familial risks and prediction performance. Thus, the model with age, sex, and pack-years was analyzed further.

Next, we tested the role of genetic variants to investigate whether SNPs can improve prediction performance of COPD status. To incorporate a large number of SNPs for polygenic prediction, we applied penalized regression with a number of BLUP-filtered SNPs using un-penalized age, sex, and pack-years. Three ethnic-specific prediction models with varying numbers of SNPs and penalties were developed and their prediction performance was evaluated using AUC and 5-fold cross validation (Table 3). Models using ridge penalty generally out-performed those with other penalties. The highest AUC, depicted in bold in Table 3, was achieved with ridge penalty within each population.

**TABLE 3:** Prediction with BLUP-filtered SNPs (in AUC).

Penalty*	Number of SNPs**	**AA model AUC**	**EA model AUC**	**NHW model AUC**
Ridge	0.1k	**0.749 (0.0368)**	**0.786 (0.0244)**	0.746 (0.0116)
	0.5k	0.743 (0.0391)	0.786 (0.0244)	0.743 (0.0155)
	1k	0.731 (0.0337)	0.786 (0.0244)	0.721 (0.0148)
	5k	0.739 (0.0363)	0.786 (0.0244)	0.751 (0.0113)
	10k	0.742 (0.0345)	0.783 (0.0266)	0.754 (0.0127)
	15k	0.741 (0.0359)	0.783 (0.0266)	0.753 (0.0105)
	20k	0.742 (0.0354)	0.783 (0.0265)	0.75 (0.0095)
Lasso	0.1k	0.712 (0.0342)	0.784 (0.0253)	0.735 (0.0132)
	0.5k	0.683 (0.0376)	0.784 (0.0253)	0.703 (0.0108)
	1k	0.681 (0.0403)	0.784 (0.0253)	0.679 (0.0128)
	5k	0.679 (0.03)	0.784 (0.0253)	0.671 (0.0061)
	10k	0.684 (0.0287)	0.784 (0.0255)	0.694 (0.011)
	15k	0.695 (0.0314)	0.784 (0.0255)	0.704 (0.0077)
	20k	0.7 (0.0323)	0.784 (0.0255)	0.699 (0.001)
Elastic net	0.1k	0.712 (0.0342)	0.784 (0.0253)	0.735 (0.0131)
	0.5k	0.683 (0.0376)	0.784 (0.0253)	0.703 (0.0108)
	1k	0.681 (0.0403)	0.784 (0.0253)	0.679 (0.0128)
	5k	0.679 (0.03)	0.784 (0.0253)	0.671 (0.0061)
	10k	0.684 (0.0287)	0.784 (0.0254)	0.694 (0.011)
	15k	0.695 (0.0314)	0.784 (0.0255)	0.704 (0.0077)
	20k	0.7 (0.0323)	0.784 (0.0255)	0.699 (0.001)

* All penalized models include Age, Sex, and Pack-years as covariates. ** SNPs with the largest absolute BLUP value were prioritized.

We conducted the same analysis with SNPs prioritized by *p*-value of logistic regression (Supplementary Table S2). However, the BLUP approach showed better performance. Results with other performance parameters, such as sensitivity and specificity, showed a similar pattern with AUC.

### External Validation With NHW Population

The primary focus of this study was to observe the differences of genetic prediction of COPD risk in different ethnic groups. To determine whether the differences are valid, we applied the three best prediction models for each ethnic group to an independent dataset. If the prediction performance of specific ethnic model was higher than the other two, it might suggest the necessity of ethnic-specific COPD studies.

We tested the performance of each model using a NHW sample in MESA study (due to the limited access to the MESA dataset). We first applied the model with the best AUC to each ethnic group: 100 SNPs for AA and EA, and 10,000 SNPs for NHW. Due to the genotyping platform difference, targeted imputation was performed for the MESA dataset. Not all imputed SNPs, however, could pass the quality controls (Table 4). With the applicable SNPs, the best AUC was observed with the NHW model (Table 4). Since the number of SNPs in NHW model was larger than in the other two, we used BLUP-filtered top 100 SNPs for all models and repeated the analyses. In both cases, the best AUC was observed with NHW model (Table 4). Because of limited access to the MESA dataset, only one of the three ethnicities modeled was validated. Because of this restriction, the original dataset was used for cross-ethnicity prediction ability by applying ethnic-specific models to the other ethnic groups. Similar to the result with MESA dataset, the best performance of each ethnic model was observed when performed pairwise application of ethnic-specific models to the other ethnic groups in the original dataset (Supplementary Table S3). It would give some idea about necessity for cross-ethnic prediction, but more careful further analyses with external validation datasets are needed for confidence.

**TABLE 4:** External validation of three ethnic models on NHW population.

**Population Modeled**	**Covariates**	**Number of SNPs modeled**	**AUC**
African American	Age, Sex, Pack-years	70/100 (Best model*)	0.643
		Top 100 (BLUP**)	0.643
East Asian		83/100 (Best model*)	0.700
		Top 100 (BLUP**)	0.700
Non-Hispanic White		7841/10000 (Best model*)	0.711
		Top 100 (BLUP**)	0.721

* SNPs selected in the best performed model in Table 3. ** SNPs with BLUP filtering.

### Variability Explained by Clinical Covariates and SNPs

To estimate the variability associated with each variable, we investigated the best model in each ethnic category. As described in the “Materials and Methods” section, we re-fitted the best model with whole samples in each population and evaluated the residual deviance of each variable (Table 5). Notably, the largest portion of total variability was unexplained in all ethnic groups, indicating that the majority of disease susceptibility still remains unexplained. The tiny fraction of variance explained by SNPs (except in NHW) was striking, although it might be due to the small number of SNPs in the model. Age explained a substantial proportion of variability in all ethnicities, but the contribution of other covariates was highly variable across each ethnicity. Note also that the variance explained in NHW was far large. One possible interpretation of the bias in% variance explained by SNPs for NHWs is that the SNP could be larger SNP selection in NHW population, reduced LD in AA population, and lower power for the EA cohort study, and so one. However, it is not yet clear whether this was due to the issues with the study design, or due to cultural or ethnic differences.

**TABLE 5:** ariability in COPD explained by clinical covariates and SNPs.

	**AA**	**EA**	**NHW**
Unexplained	88.45%	88.05%	65.86%
Age	11.27%	8.65%	3.74%
Sex	0%	1.78%	0%
Pack-years	0.27%	0.13%	6.06%
SNPs	0.01%	1.39%	24.34%

## Discussion

Although the possibility of ethnicity influencing COPD susceptibility is appreciable since genetic susceptibility variants might be different across ethnicities, little information is available concerning between ethnicities difference in genetic risk for COPD. Only a few studies have noted the role of ethnic differences in COPD development (Hansel et al., 2013; Gilkes et al., 2017), and investigated genetic differences of COPD susceptibility across ethnicities (Zhou et al., 2013), whereas none have developed COPD prediction models using ethnicity-specific loci. Many studies have demonstrated a large number of genetic risk loci being shared across ethnicities (Benyamin et al., 2017). However, our study indicates that different ethnic groups with different genetic architecture may have substantial impact on the accuracy of different prediction models.

Here, we investigated ethnicity-based genetic differences in COPD development by building and evaluating prediction models with three different ethnic groups. We discovered ethnicity-specific genetic risk factors, using both BLUP from a mixed model and *p*-values from the linear model. Because COPD is a complex disease and many genetic loci with small effect size are likely to be involved in developing disease, we paid our attention to comparing prediction performance as a combined effect from 20,000 SNPs in each ethnic group, instead of focusing on their individual statistics. The first interesting observation was made when between ethnicities and within one ethnicity prediction models were compared. A remarkable number of SNPs overlapped within one ethnicity (although none of the samples overlapped) compared to those between ethnicity. Moreover, we found BLUP models to have more overlapped SNPs in both within and between ethnicities than *P*-value models. Moreover, the prediction models with BLUP-filtered SNPs showed relatively higher AUC values compared to those with *p*-value-filtered SNPs. BLUP-filtered SNPs have a number of advantages of BLUP selection, such as accounting for relationship matrix and handling unbalanced designs.

The proposed methodological framework for ethnicity-specific prediction of COPD can enhance the interpretation of results from validation studies. A number of studies have been attempted to refine the interpretation of validation study results by distinguishing between model reproducibility and model transportability (Debray et al., 2015). Model reproducibility refers to model performance across new samples from the same target population, which can be approximated with resampling techniques such as cross-validation. Transportability refers to model performance across samples from different but related source populations and can only be assessed in external validation studies. In this study, we performed both validation studies: cross-validation for internal validation and a comparison with a completely different dataset for external validation.

There were several limitations in our study. Although our cross-validation results were generally consistent with those of other studies, demonstrating the challenges of cross-ethnicity prediction, each population dataset used in this study had differences in study designs, genotyping arrays, and sample sizes, specially with EA population. The number of COPD in EA population is small, and thus it is likely lead to low power for examining SNP effects for this population. Although we observed cross-ethnicity prediction ability with the original dataset, there were not AA and EA subjects available for external validation. It would be more compelling if each ethnic-specific prediction model predicted best in the corresponding ethnic dataset for external validation. Also, the factions of variance explained in AA and EA subjects were relatively small than that in NHW. This might be partly due to the amount on imputation. Because of platform difference, a large number of imputation SNPs in EA group was analyzed. To make sure our imputation dataset applicable in our study, we checked the imputation performance by measuring concordance rate with varying imputation threshold and the overall concordance rate (about 95%) was tolerable (Supplementary Figure S4). However, there still remain questions like “were larger fractions of variance explained in AA and EA subjects if larger numbers of SNPs were used?” or “is it also possible that the most important SNPs are not being identified in EA and AA subjects due to the smaller numbers of cases in those samples?.” We used AUC as a measure of performance and choice for the best model, but in some cases the differences in AUC were not statistically significant. However, the main aim of this study was to appreciate the necessity of considering ethnic differences in COPD risk. Evidence from this study complements those from others and supports substantial ethnic-specific differences in COPD susceptibility. Understanding these differences might be particularly important in preventing of COPD as well as developing therapeutic strategies and identifying molecular treatment targets.

## Data Availability Statement

Publicly available datasets were analyzed in this study. This data can be found here: http://www.copdgene.org/.

## Ethics Statement

This study was carried out in accordance with the recommendations of exempted deliberation, the Seoul National University Institutional Review Board (IRB) with written informed consent from all subjects. All subjects gave written informed consent in accordance with the Declaration of Helsinki. The protocol was approved by the Seoul National University IRB.

## Author Contributions

JG and SW designed and directed the project, interpreted the results, and finalized the manuscript. JA performed the genotype imputation. JG analyzed the data and wrote initial manuscript. JS, ES, and MC provided critical feedback and helped shape the research, analysis and manuscript. SW supervised the project.

## Conflict of Interest

The authors declare that the research was conducted in the absence of any commercial or financial relationships that could be construed as a potential conflict of interest.
