# Supplementary material for: A Between Ethnicities Comparison of Chronic Obstructive Pulmonary Disease Genetic Risk
Source: Front Genet. 2020 Apr 21;11:329. doi: 10.3389/fgene.2020.00329 (PMC7187688; doi:10.3389/fgene.2020.00329)
Supplement: Supplementary file 1 [file Table_1.docx]

Table S1 Population-specific estimates of SNP heritability

| Project | Population | Full data | Full data with smoking status |
| --- | --- | --- | --- |
| COPDGene | African American (AA) | 0.349 (0.109) | 0.314 (0.105) |
|  | Non-Hispanic White (NHW) | 0.414 (0.053) | 0.414 (0.053) |
| KARE | East Asian (EA) | 0.164 (0.032) | 0.158 (0.032) |
