# Supplementary material for: A Between Ethnicities Comparison of Chronic Obstructive Pulmonary Disease Genetic Risk
Source: Front Genet. 2020 Apr 21;11:329. doi: 10.3389/fgene.2020.00329 (PMC7187688; doi:10.3389/fgene.2020.00329)
Supplement: Supplementary file 2 [file Table_2.docx]

Table S2 Prediction with p-value-filtered SNPs (in AUC)

| Models* | # of SNPs** | African American | East Asian | Non-Hispanic Whites |
| --- | --- | --- | --- | --- |
| Ridge | 0.1k | 0.719(0.0252) | 0.785(0.0238) | 0.741(0.0091) |
|  | 0.5k | 0.719(0.0219) | 0.779(0.0235) | 0.723(0.0063) |
|  | 1k | 0.722(0.0253) | 0.773(0.0233) | 0.721(0.0055) |
|  | 5k | 0.74(0.032) | 0.762(0.0244) | 0.75(0.007) |
|  | 10k | 0.738(0.0335) | 0.783(0.0265) | 0.748(0.0067) |
|  | 15k | 0.736(0.0344) | 0.783(0.0264) | 0.748(0.0061) |
|  | 20k | 0.735(0.0344) | 0.782(0.0267) | 0.746(0.0051) |
| Lasso | 0.1k | 0.719(0.0244) | 0.784(0.0253) | 0.738(0.0071) |
|  | 0.5k | 0.713(0.0243) | 0.784(0.0253) | 0.712(0.0081) |
|  | 1k | 0.711(0.0217) | 0.784(0.0253) | 0.706(0.0086) |
|  | 5k | 0.693(0.0297) | 0.784(0.0253) | 0.711(0.0088) |
|  | 10k | 0.698(0.0269) | 0.784(0.0255) | 0.704(0.0088) |
|  | 15k | 0.695(0.028) | 0.784(0.0255) | 0.702(0.0056) |
|  | 20k | 0.696(0.0397) | 0.784(0.0255) | 0.703(0.0109) |
| Elastic  net | 0.1k | 0.719(0.0239) | 0.784(0.0253) | 0.738(0.0072) |
|  | 0.5k | 0.713(0.0243) | 0.784(0.0253) | 0.712(0.0083) |
|  | 1k | 0.711(0.0217) | 0.784(0.0253) | 0.706(0.0086) |
|  | 5k | 0.693(0.0297) | 0.784(0.0253) | 0.711(0.0088) |
|  | 10k | 0.698(0.0269) | 0.784(0.0255) | 0.704(0.0088) |
|  | 15k | 0.695(0.028) | 0.784(0.0254) | 0.702(0.0056) |
|  | 20k | 0.696(0.0397) | 0.784(0.0255) | 0.703(0.0109) |

*All penalized models include Age, Sex, and Pack-year as covariates

**SNPs with the smallest p-value were prioritized
