# Supplementary material for: A Between Ethnicities Comparison of Chronic Obstructive Pulmonary Disease Genetic Risk
Source: Front Genet. 2020 Apr 21;11:329. doi: 10.3389/fgene.2020.00329 (PMC7187688; doi:10.3389/fgene.2020.00329)
Supplement: Supplementary file 3 [file Table_3.docx]

Table S3 Internal validation of three ethnicity models by pairwise application

| Best prediction model | Validation population | | |
| --- | --- | --- | --- |
|  | African American | East Asian | Non-Hispanic Whites |
| African American | **0.751** | 0.698 | 0.708 |
| East Asian | 0.710 | **0.771** | 0.721 |
| Non-Hispanic White | 0.692 | 0.713 | **0.762** |
