# Supplementary figures and images for: A Between Ethnicities Comparison of Chronic Obstructive Pulmonary Disease Genetic Risk
Source: Front Genet. 2020 Apr 21;11:329. doi: 10.3389/fgene.2020.00329 (PMC7187688; doi:10.3389/fgene.2020.00329)

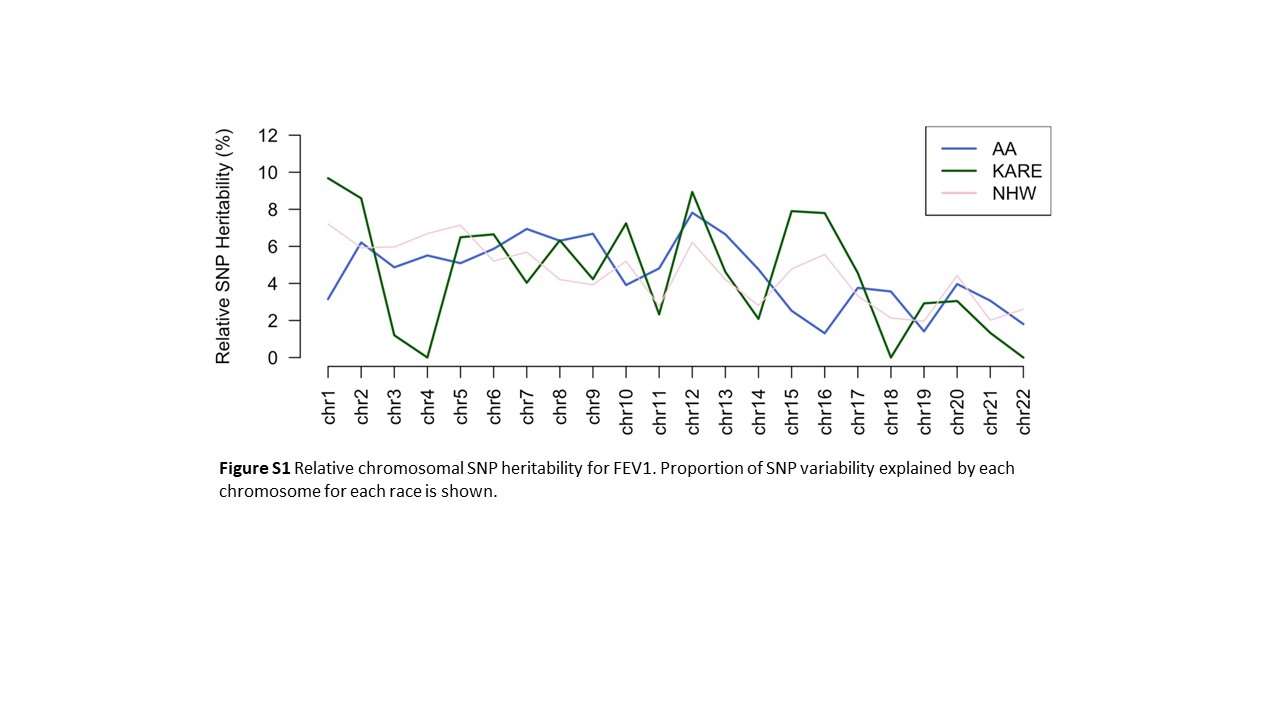

Supplement: Supplementary file 4 [file Image_1.jpg]

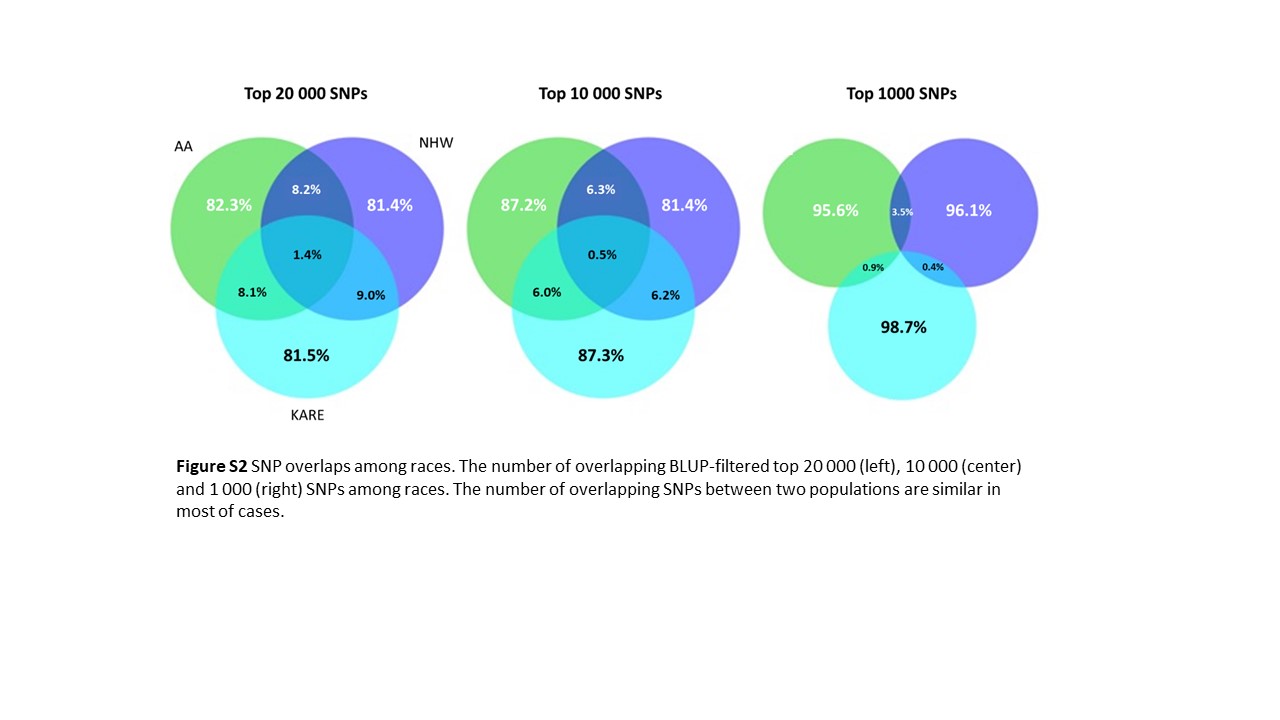

Supplement: Supplementary file 5 [file Image_2.jpg]

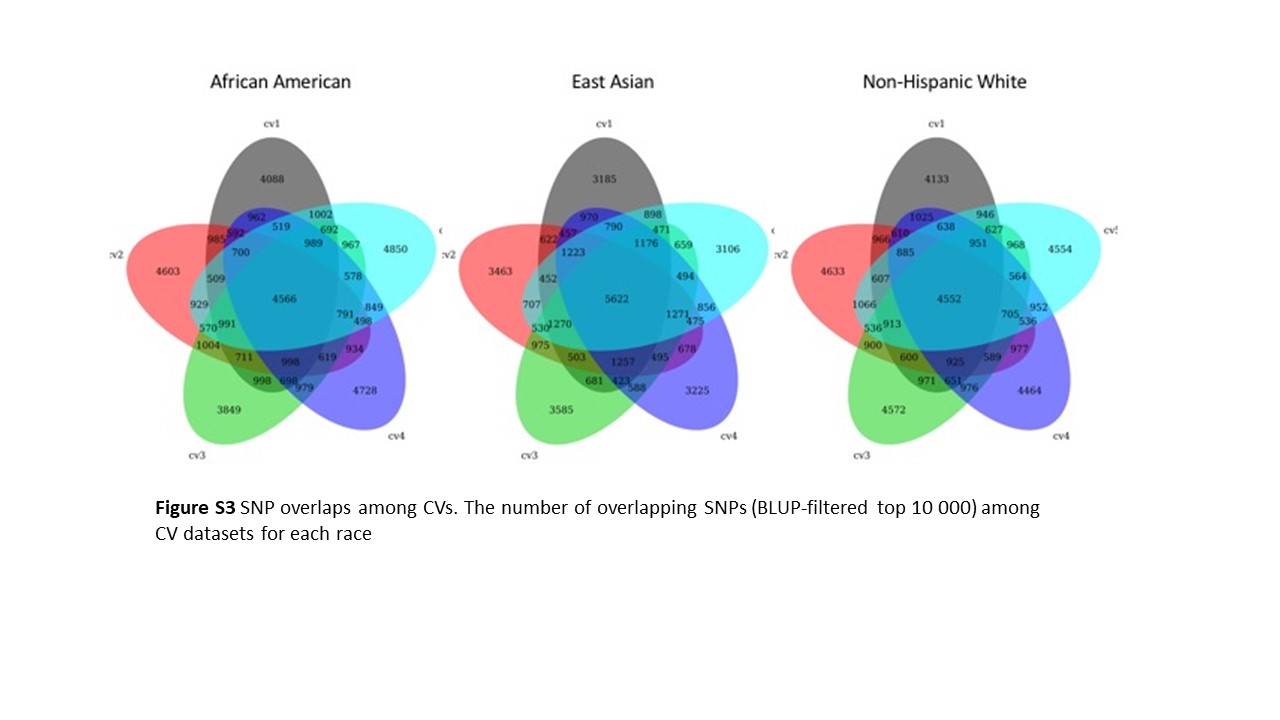

Supplement: Supplementary file 6 [file Image_3.jpg]

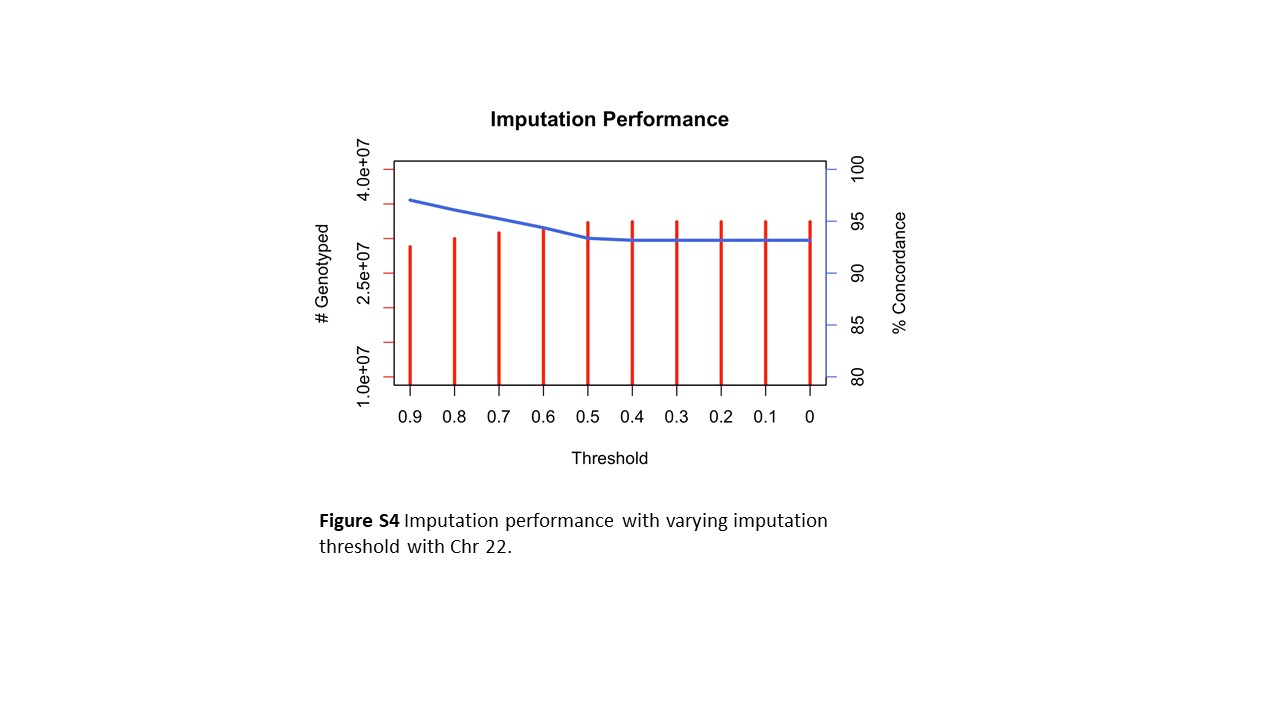

Supplement: Supplementary file 7 [file Image_4.jpg]
